# Quantifying the Resilience of Coal Energy Supply in China Toward Carbon Neutrality

**DOI:** 10.34133/research.0398

**Published:** 2024-07-16

**Authors:** Yongzheng Sun, Guanghui Wen, Haifeng Dai, Yu Feng, Sandro Azaele, Wei Lin, Fubao Zhou

**Affiliations:** ^1^ School of Mathematics, China University of Mining and Technology, Xuzhou 221116, China.; ^2^ School of Safety Engineering, China University of Mining and Technology, Xuzhou 221116, China.; ^3^ School of Mathematics, Southeast University, Nanjing 210096, China.; ^4^ School of Cyber Science and Engineering, Southeast University, Nanjing 210096, China.; ^5^ China Coal Transportation and Distribution Association, Beijing 100160, China.; ^6^ Department of Physics and Astronomy “G. Galileo”, University of Padova, Via F. Marzolo 8, Padova 35131, Italy.; ^7^ Research Institute of Intelligent Complex Systems, School of Mathematical Sciences, LMNS, and SCMS, Fudan University, Shanghai 200433, China.; ^8^ MOE Frontiers for Brain Science, Shanghai 20032, China.; ^9^ Shanghai Artificial Intelligence Laboratory, Shanghai 200232, China.

## Abstract

Facing the challenge of achieving the goal of carbon neutrality, China is decoupling the currently close dependence of its economy on coal use. The energy supply and demand decarbonization has substantial influence on the resilience of the coal supply. However, a general understanding of the precise impact of energy decarbonization on the resilience of the coal energy supply is still lacking. Here, from the perspective of network science, we propose a theoretical framework to explore the resilience of the coal market of China. We show that the processes of increasing the connectivity and the competition between the coal enterprises, which are widely believed to improve the resilience of the coal market, can undermine the sustainability of the coal supply. Moreover, our results reveal that the policy of closing small-sized coal mines may not only reduce the safety accidents in the coal production but also improve the resilience of the coal market network. Using our model, we also suggest a few practical policies for minimizing the systemic risk of the coal energy supply.

## Introduction

As the world’s second-largest economy and a predominant consumer of coal, accounting for over 50% of global demand, China also stands as the largest emitter of carbon dioxide (CO_2_), exerting a significant global influence both economically and environmentally [1–4]. While transitioning the economy away from coal dependency has emerged as a crucial development strategy, expected to result in a peak in China’s coal consumption, coal currently accounts for over 60% of the total fuel consumption for China’s electricity production, maintaining its dominant position in the country’s energy supply [1,5–7].

The COP28 conference, hosted in Dubai, successfully culminated in the first global assessment of climate action as stipulated by the Paris Agreement [8]. This event calls for the implementation of a structured shift away from fossil fuel dependency toward carbon-neutral energy solutions. Since coal is a predominant source of China’s CO_2_ emissions, accounting for 75% in 2020 [9], China’s coal industry is increasingly facing international and domestic pressures to reduce carbon emissions [1]. In response to these pressures and in recognition of its responsibility in global climate mitigation, the Chinese government has set ambitious long-term goals, aiming to reach a carbon emissions peak by 2030 and achieve carbon neutrality by 2060. Facing the challenge of CO_2_ emissions reductions, China has implemented measures to foster a transition from patterns of excessive energy consumption and high CO_2_ emissions to a more sustainable trajectory [10–13] (see Fig. S1). Key steps in reducing coal use involve closing coal mines, imposing limits on coal consumption in certain regions, and promoting the deployment of clean energy sources [1,3,14]. Existing studies primarily focus on the effects of renewable energy and eco-innovation on carbon emissions [12,13,15,16]. As far as we know, there are no theoretical studies that explore how China’s energy decarbonization efforts affect the resilience of its energy supply. Therefore, it is necessary to study the resilience of China’s coal market in the context of carbon neutrality.

The concept of resilience in complex systems was first introduced by Holling to characterize an ecosystem’s ability to maintain its functionality in the face of perturbations [17]. Over the past couple of decades, research has predominantly focused on understanding the resilience of complex ecosystems [18–21]. It is only in recent years that significant attention has turned toward exploring the resilience of various economic systems, such as banking systems [22–24], global production networks [25–27], labor markets [28], and energy systems [29]. Complex systems may encounter a tipping point, beyond which their functionality deteriorates or collapses [30–36]. However, predicting the tipping point of resilience loss in such systems is challenging due to their large number of components. Several dimension-reduction approaches have been proposed to transform high-dimensional complex systems into effective low-dimensional systems [37–40]. Quantitative analysis of the simplified system enables the capture of key factors influencing the resilience of the original complex system [38,41,42]. More recently, a general dimension reduction approach, introducing a scaling factor for recovery rates, provides accurate estimates of the distance to tipping points across mutualistic systems [39]. This method has shown promising performance in predicting tipping points in complex systems.

Similar to a variety of complex systems, the coal supply system can also experience sudden regime shift from normal function to system collapse, often passing through a tipping point. This potential loss of resilience in the coal market will pose a threat to China’s energy security. Previous studies on the resilience of energy supply markets have often treated the market as a whole, neglecting the intricate interactions among energy enterprises [43]. Some research endeavors have also attempted to explore the causal relationships among external factors such as clean energy consumption, financial globalization, industrialization, and carbon emissions [44,45]. Therefore, it is necessary to develop a more comprehensive framework capable of quantifying the impact of the complex interactions among energy enterprises and policy perturbations on the resilience of energy supply markets.

Here, we propose a simple and efficient framework to explore the resilience of China’s coal market from the viewpoint of network science. Regarding the coal market as an energy ecosystem, we construct China’s coal market network from transaction data between coal enterprises and power plants. Utilizing dimension-reduction methods and bifurcation analysis, we quantitatively investigate how network structures and several realistic perturbations, including the shutdown of coal mines, cancellation of coal trade contracts, and changes in market environments, influence the resilience of the coal market reconstructed from empirical data. We demonstrate, both analytically and numerically, that the proposed model can forecast the loss of resilience in the coal market. Astonishingly, we find that connectivity leads to a loss of resilience, contrary to its widely believed role in enhancing resilience for the coal market. In particular, we show that key steps toward China’s carbon neutrality, such as the closure of small-scale coal mines and the promotion of regional low-carbon development, are effective and practical strategies for sustaining the resilience of the coal energy supply. Our study significantly improves our understanding of the resilience of the energy supply market. To our knowledge, this is the first study to examine the resilience of the energy market from the perspective of network science. Our approach effectively captures the systemic risks arising from mutual competition within the coal supply market, offering valuable insights for the systemic study of other attribute-like energy markets.

## Results

### A general framework for quantifying coal market network resilience

In China, the distribution of coal resources is highly uneven, with most power plants, particularly those along the southeastern coast, relying on coal imports from the major coal-producing regions in northern China. In 2018, the raw coal production in 3 provinces—Shanxi, Shaanxi, and Inner Mongolia—comprised 66.4% of China’s total production [6]. However, the majority of the top 10 provinces in terms of installed thermal power capacity do not correspond to the major coal-producing regions. This disparity highlights the fact that the majority of coal (approximately 75%) produced in coal-producing regions across the country is transported to other areas, naturally creating a network structure within China’s coal market. Building upon existing research that has identified the substantial influence of network structures on the resilience of ecosystems [18,19,25], financial networks [22–24], global food trade networks [25–27], and energy systems [29], it is imperative to address the risk of resilience loss in the coal market from a network perspective, rather than solely focusing on individual coal mines.

**Fig. 1.**
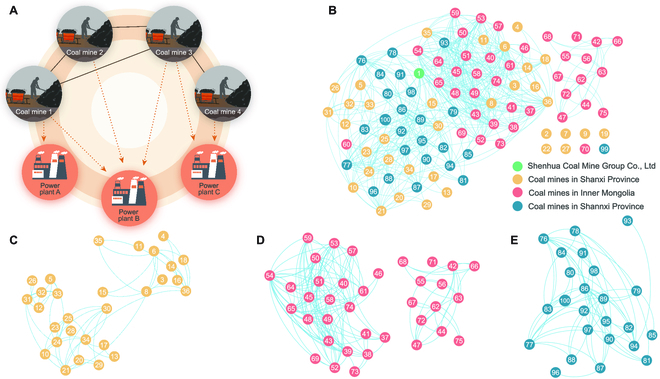
Coal market network in China’s major coal production areas. (A) A schematic for coal market network constructed from the bipartite network of coal mines and thermal power plants. For those 2 coal mines supplying coal to the same thermal power plant, a link between these two coal mines exists. Regarding each coal mine as a node, and the link between coal mines as edges, then a coal market network can be built. (B) China’s coal market network is built according to the strategy in (A). Here, different colors represent coal mines in different provinces. The network consists of 100 coal mines that account for over 60% total coal output in China, and 6 of which are isolated nodes. (C to E) The coal market network of Shanxi, Inner Mongolia, and Shannxi provinces.

We consider a coal market network of *N* components that represent coal mines or coal enterprises (in the following, coal mines for brevity). The coal mine *i* has a time-dependent coal production *R_i_*(*t*), which is influenced by the coal mine *j*, connected to *i* through a network of interactions, denoted by a weighted adjacency matrix *C* = (*C_ij_*). This matrix is defined as follows: for *i*, *j* = 1, 2, …, *N*, *C_ij_* > 0 if coal mines *i* and *j* supply coal for the same thermal power plant; otherwise, *C_ij_* = 0 (see Fig. 1).

In general, the dynamics of the coal production of each mine include 2 processes, viz., the intrinsic growth and the external market competition, which then obey

$$\frac{{\mathrm{dR}}_{i}}{\mathrm{dt}}=\alpha_{i}R_{i}\left(R_{i}-A_{i}\right)\left(1-\frac{R_{i}}{K_{i}}\right)-\delta \sum_{j=1}^{N}\;C_{\mathrm{ij}}R_{i}\;f_{i}\left(R_{j}\right).$$ (1)

Here, the first term on the right-hand side of system (1) is known as the logistic growth with an intrinsic growth rate *α_i_* > 0 and a carrying capacity *K_i_* (the maximal production ability of coal mines), and the parameter *A_i_* stands for the Allee effect, according to which, for a low production (*R_i_* < *A_i_*), the coal production renders in a manner of negative growth. Moreover, the second term integrates all the influences of coal mines connected to coal mine *i*, where the function *f_i_*(*R_j_*) depicts how coal mine *j* impacts *i* through the market competition. Throughout, we assume that *f_i_*(*R_j_*) = *θ_ij_R_j_* with *θ_ij_* an inflow rate parameter. The depletion coefficient *δ* quantifies the strength of network interaction, where *δ*= 0 indicates the absence of any such interactions.

The model (1) is based on an understanding of the dynamics of the coal supply system and the foundational principles of network science. The cubic growth function in model (1) accommodates the existence of a stable sustainable state $R_{i}^{H}$, an unstable low-resilience state $R_{i}^{L}$, and a depleted state *R_i_* = 0. This function has been successfully applied to model the dynamics of natural resources [26] and ecosystems [37]. By leveraging the foundational tenets of network science, the second term captures the competitive dynamics among coal mines through trade networks, which influence production outcomes. Despite its apparent simplicity, this model demonstrates its capacity to unveil the core characteristics and dynamic behaviors of the coal market network. It offers significant advantages in elucidating the key influencing factors of the resilience of the coal market, thereby providing valuable insights into this complex system.

Previous studies about the resilience of ecological networks show that the connectivity and average interaction strength have crucial impact on the resilience of ecosystems [18,19]. Akin to the ecological network, a loss of system (1)’s resilience can be induced by the changes of the intrinsic growth parameters and by the *N*^2^ elements in the network adjacency matrix *C*. To make it feasible to numerically calculate and mathematically analyze the resilience of the coal market network, we extend the dimension reduction framework proposed by Gao et al. in [37] to transform the high-dimensional system into the effective 1-dimensional (1-D) model that not only captures the essential resilience of the high dimensional system but also predicts precisely the onset of the tipping point of ecological networks [37,38,41], even in the presence of noisy perturbations [42].

Applying the dimension reduction framework (see Methods) to system (1) yields an effective 1-D system as

$$\begin{array}{cc} \frac{{\mathrm{dR}}_{\text{eff}}}{\mathrm{dt}}= & -\alpha_{\text{eff}}A_{\text{eff}}R_{\text{eff}}+\left(\alpha_{\text{eff}}+\frac{\alpha_{\text{eff}}A_{\text{eff}}}{K_{\text{eff}}}\right)R_{\text{eff}}^{2}\\ & -\frac{\alpha_{\text{eff}}}{K_{\text{eff}}}R_{\text{eff}}^{3}-{\delta \eta }_{\text{eff}}R_{\text{eff}}^{2}, \end{array}$$ (2)

where *R*_eff_ is the effective average coal production of the whole coal market, *η*_eff_ = *θ*_eff_*β*_eff_ with the macroscopic parameter *β*_eff_ characterizing the combined effect of the microscopic description *C_ij_* of the inter competition. In addition, *α*_eff_, *A*_eff_, *K*_eff_, and *θ*_eff_ account for the effective growth rate, the Allee effect, the carrying capacity, and the inflow rate, respectively, when averaged across the network structure.

**Fig. 2.**
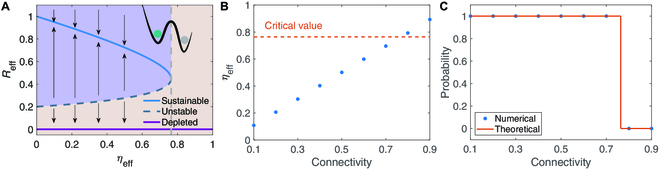
Network connectivity and resilience of coal market networks with ER random topologies. (A) Bifurcation diagram for the steady states $R_{\text{eff}}^{H,L,*}$ of the reduced system (2) as the parameter *ta*_eff_ changes. Here, the loci of the resilience loss is calculated using Eq. 3 as $\eta_{\text{eff}}^{c}=0.76$ for the parameters specified in the main text. (B) Monotonic change of the bifurcation parameter *η*_eff_ with the connectivity parameter *p*. (C) The probability of system (1) exhibiting a sustainable state changes with the connectivity of the ER random topologies containing *N* = 100 nodes. The elements *C_ij_* in the topologies are randomly taken from the Gaussian normal distribution *N*(*μ*, *σ*^2^) with *μ* = 0.2 and *σ* = 0.04. The displayed results are calculated through averaging 100 realizations and the initial states are randomly selected near the steady state $R_{\text{eff}}^{H}$.

To proceed, we take *η*_eff_, a value fully determined by the network topology and the average outflow rate *θ*_eff_, as a bifurcation parameter for the 1-D reduced system (2), where a trivial solution $R_{\text{eff}}^{*}=0$ for *η*_eff_ > 0 represents the depleted state. This system also possesses a stable steady state $R_{\text{eff}}^{H}$ and an unstable steady state $R_{\text{eff}}^{L}$ when $0<\eta_{\text{eff}}<\eta_{\text{eff}}^{c}$. The state $R_{\text{eff}}^{H}$ corresponds to the sustainable state of the coal market, in which the average coal production is high. The low-production state $R_{\text{eff}}^{L}$ is unstable and may transit to the desired sustainable state $R_{\text{eff}}^{H}$ or the undesired depleted state $R_{\text{eff}}^{*}=0$. However, there is a tipping point after which the system loses its resilience, and the precise loci of the resilience loss is calculated as

$$\eta_{\text{eff}}^{c}≜\frac{\alpha_{\text{eff}}}{K_{\text{eff}}\delta_{\text{eff}}}\left(K_{\text{eff}}+A_{\text{eff}}-2\sqrt{K_{\text{eff}}A_{\text{eff}}}\right),$$ (3)

as shown in Fig. 2A (see Methods).

Clearly, an increase of *η*_eff_ induces the system to lose its resilience. Notice that the parameter *θ*_eff_ depends on the transactions between the coal mines and the thermal power plants, which need to meet the electricity demand. So, *θ*_eff_ usually changes more slowly than *R_j_*, and it is reasonably assumed to be fixed in all the simulations, i.e., *θ_ij_* = *θ*. Thus, *η*_eff_ is fully determined by the network topologies. Using the dimension reduction method, we have mapped the original system (1) into the (*R*_eff_, *η*_eff_) space. Therefore, we can predict the system’s response to the variations of the parameter *η*_eff_, where the variations could be induced by connectivity changes, node/link losses, and link weights changes, and so on [37,41]. As such, we focus on studying the influence of the network complexity measurements and a few realistic perturbations on the resilience of the coal market network.

The resilience of the coal market network may be influenced by various factors such as network topology, node dynamics, coupling methods and interaction strengths. In the following, we place particular emphasis on the influence of network topology including network connectivity, heterogeneity, and modularity.

### Influence of network connectivity

Connectivity, an indicator that describes how well parts of the network connect to one another, has an important impact on the stability of complex networked systems [18,19,46]. This impact is influenced by many factors such as node dynamics, network topology, and the nature and intensity of interactions among components. Bardoscia et al. [24] reported that connectivity can undermine the stability of financial systems by creating cyclical structures that amplify financial distress. Recent studies on the sustainability of natural resources have shown that a network’s interconnectedness may increase or decrease its resilience, depending on the network structure [26].

Here, we use some generic networks to construct the networks for the coal market. Specifically, we take *α_i_* ≡ 0.5, *A_i_* ≡ 0.2, *K_i_* ≡ 1, *θ*= 0.95, and *δ*= 0.2, so that, as shown in Fig. 2A, the reduced system (2) has a tipping point $\eta_{\text{eff}}^{c}=0.76$, after which its resilience is lost. To demonstrate the efficacy of the dimension reduction method in our coal market system, we performed numerical simulations for the original system (1) with the Erdős–Rényi (ER) random topologies containing *N* nodes. We assume that any 2 nodes are connected with the probability *p*, and the weights are randomly taken from the standard Gaussian normal distribution *N*(*μ*, *σ*^2^) with *μ* > 0. Depending on *μ* and *σ*, the distribution has to be truncated to avoid negative weights and the truncated distribution shifted to keep its mean value equal to *μ*. We can see from Fig. 2B that as the connectivity *p* increases, *η*_eff_ increases. The probability that the system exhibits a resilient state (approaching a stable steady state $R_{\text{eff}}^{H}$) as a function of the connectivity is shown in Fig. 2C. Clearly, there is a transition from the resilient state to the nonresilient state (undesirable depleted state) when the connectivity exceeds the critical point which corresponds to the tipping point of $\eta_{\text{eff}}^{c}$. Although $\eta_{\text{eff}}^{c}$ obtained in (3) is fully determined by the node dynamics and is independent of the network topology, the resilience loss strongly depends on the connectivity of the underlying networks. The impact of connectivity on the resilience of coal market network with the Watts–Strogatz small-world [47] and Barabási–Albert scale-free [48] topologies are further demonstrated in Supplementary Materials (see Figs. S2 and S3). All these results reveal a negative role of the interconnectedness in sustaining the resilience of the coal market network, which is different from the existing results on the resilience of global resources network and labor market [26,28]. In fact, coal is a kind of bulk product with high transportation costs restricting the flow of coal between regions. Compared with other resource networks, the real coal market network has lower connectivity, which is beneficial to sustaining its resilience.

### Influence of network heterogeneity and modularity

**Fig. 3.**
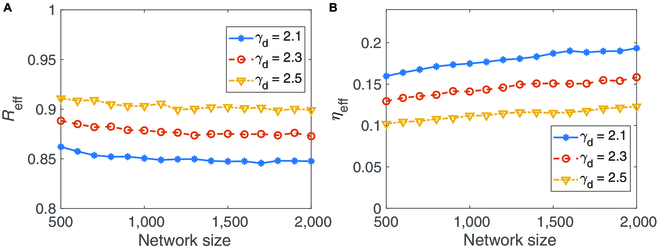
Impact of network size and heterogeneity on the resilience of coal market networks. (A) *R*_eff_ changes with the network size under different *γ_d_*. (B) *η*_eff_ changes with the network size under different *γ_d_*. Here, the Barabási–Albert scale-free networks with different power exponent *γ_d_* are used. The other parameters are the same as those used in Fig. 2. Each result is obtained through averaging 100 realizations with the initial condition randomly selected near the sustainable state.

Real-world networks are often highly heterogeneous, which affects significantly the dynamic performance of complex networks. China’s coal market network is heterogeneous due to the uneven geographical distribution of coal resources; see also the degree distribution in Fig. S4. So, we explore the influence of the network heterogeneity on the resilience of the coal market network. Using the method outlined in [49], we generate several scale-free networks with different values of the power exponent *γ_d_*. As shown in Fig. 3A, the network size has a weak impact on the network resilience. Unlike the case of the homogeneous random networks, for any given network size, the lager the exponent *γ_d_* for the coal market network, the larger the desirable steady state *R*_eff_ becomes. This clearly indicates that heterogeneity is beneficial to the resilience of the coal market network. This can be demonstrated by the results on the effect of network heterogeneity with respect to the parameter *η*_eff_; see Fig. 3B.

**Fig. 4.**
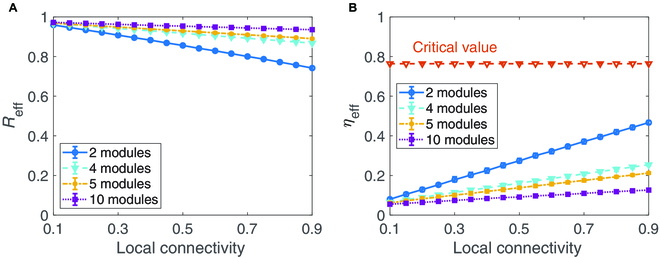
Impact of modularity and local connectivity on the resilience of coal market networks. (A) *R*_eff_ changes with the local connectivity and modules. (B) *η*_eff_ changes with the local connectivity and modules. Here, each modular is an ER random network with the same size and local connectivity, and the total size *N* = 100. The connectivity among modules is taken as 0.1, and the other parameters are the same as those used in Fig. 2. Each result is obtained through averaging 100 realizations with the initial condition randomly selected near the sustainable state.

Modularity that measures the strength of division of a network into modules (or communities), has substantial importance in maintaining the function of social and economical systems [20,23,26]. Nodes within modules have much more frequent interactions than those in different modules. As shown in Fig. 1 and also in Fig. S5, the empirical coal market network exhibits in a highly modular manner. Thus, we turn to explore the impact of modularity on the resilience of the coal market network. By generating modular networks with different numbers of modules (see Methods), Fig. 4A shows the effective mean coal output as a function of the local connectivity (the connectivity of each module). As displayed in Fig. 4B, the networks with higher modularity have smaller bifurcation parameter *η*_eff_, which thus implies that the modularity is beneficial to sustaining the resilience of the coal market network. Additionally, for a fixed number of modules, increasing local connectivity can decrease the resilience of the coal market network, which is similar to the results shown in Fig. 2 and in Figs. S2 and S3 of Supplementary Materials.

### Resilience of China’s coal market network

To achieve the goal of carbon neutrality, China needs to change its energy supply structure and implement policies to decarbonize the energy supply and demand [39], including reducing coal consumption and substantially increasing the share of new or renewable energy [4,50]. Behind the energy decarbonization are profound changes in the coal market, such as the closures of coal mines and the transaction cancellation due to regional low-carbon development, and the depression of the whole coal market caused by the energy demand decarbonization. Indeed, these realistic perturbations of the coal market can be mimicked by the changes of *N*^2^ parameters *C_ij_* in the adjacency matrix. For instance, the closures of coal mines can be simulated by removing the nodes from the networks, and the cancellation of coal transaction can be modeled by deleting the existing links. In addition, the depression of the whole coal market may lead companies to reduce coal purchases, which can be simulated by a global decrease of link weights between coal mines.

By 2002, China had more than 25,000 small-sized coal mines with an annual output of less than 300,000 tons, accounting for 90% of China’s total coal mines and 30% of the total coal output. The problem of safety production in small-sized coal mines is serious. In 1998, the death toll from safety accidents in small-sized coal mines accounts for more than 70% death toll of all coal mines in China [6]. Since 2005, the Chinese government has begun to shut down those small-sized coal mines that do not have the conditions for safe production. Some medium coal mines have also been closed simply due to the regional low-carbon development policy [3]. Moreover, the carbon neutrality goal calls for a 28% in 2035 and then further to 59% in 2050, renewable energy in primary energy supply, which implies that more and more coal mines will be closed [3].

**Fig. 5.**
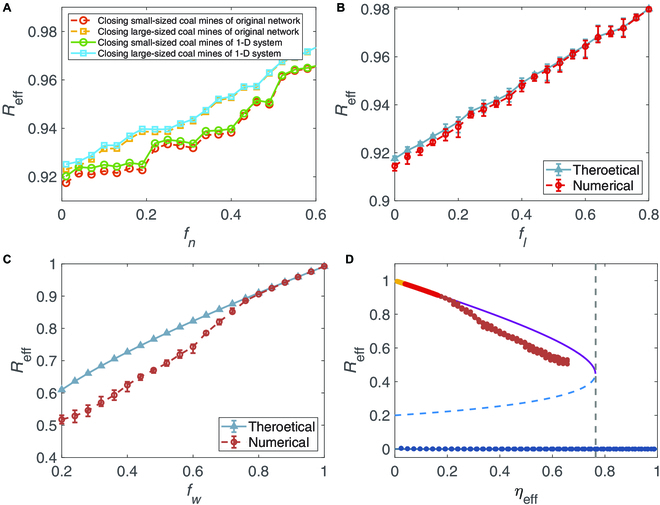
Resilience of China’s coal market network. Changes of *R*_eff_ with respect to different fractions: (A) *f_n_*, the closing fraction of the coal mines; (B) *f_l_*, the canceling fraction of the transactions between the coal mines and the thermal power plants; and (C) *f_w_*, the fraction to which all weights on average are decreased, which mimics the market depression. (D) The points of *η*_eff_ − *R*_eff_, computed using the original system and shown in (A) to (C), are well consistent with the resilience function obtained in the 1-D reduced system. The weights in (C) are taken from the Gaussian normal distribution *N*(*μ*, *σ*^2^) with *μ* = 1 and *σ* = 0.2. The weights in (A) and (D) and the other parameters are the same as those used in Fig. 2.

As such, we first explore the influence of the closures of coal mines on the resilience of the coal market network. Figure 5A displays *R*_eff_, the effective mean coal production, as a function with respect to *f_n_*, the removal fraction of the coal mines. Here, we sorted the coal mines according to their outputs. Two strategies are, respectively, adopted: Prioritizing the closures of small-sized coal mines or large-sized coal mines. As shown in the simulated results in Fig. 5A, the steady state moves in the direction of higher values of *R*_eff_ as the fraction of the closed coal mines goes up. This trend corresponds to a decrease of *η*_eff_ (see Fig. S6) and thus an increase in the resilience of the coal market network. Although closing large-sized coal mines can significantly improve the resilience of the coal market network, large-sized coal mines usually have better production conditions ensuring the safety. Thus, closing large-sized coal mines can affect the coal market supply. Hence, our findings imply that the closure of small-sized coal mines can not only decrease safety accidents but also enhance the resilience of the coal market network.

It is widely believed that the electricity demand is inevitably influenced by the variations of economy growth and seasonal changes [51]. As the main fuel consumption of China’s electricity production, the coal demand changes according to the fluctuations of economic growth and seasonal changes. This definitely leads to a cancellation or an increase of the coal transactions between the coal mines and the thermal power plants, which, as introduced above, corresponds to the removal/introduction of the links in the network. Moreover, the energy demand decarbonization toward carbon neutrality could also lead to the cancellation of long-term coal contracts between coal mines and thermal power plants. Figure 3B shows the influence of link removal, *f_l_*, on the resilience of China’s coal market network, since link removal reduces the connectivity of the network. In addition, the more competitive the market, the denser the coal market network. Thus, an overly competitive coal market network undermines the stability of coal supply, which is confirmed by the simulations in Fig. 5C, where the reduction of link weights, *f_w_*, is taken into account.

This implies that a fully competitive coal market probably undermines the stability of the coal supply and that limiting the competitions among the coal mines can enhance the resilience of the coal market. Figure 5D further demonstrates the efficacy of the 1-D reduced system. Such an efficacy is due to the fact that the above realistic perturbations lead to the variations of the bifurcation parameter *η*_eff_ for the 1-D reduced system (see Figs. S6A to C). We also use the dimension reduction method to predict the resilience loss of the coal market networks in the provinces of Shanxi, Inner Mongolia, and Shannxi (see Fig. S7) and the ER networks (see Fig. S8).

Notice that Fig. 5A to C and Fig. S1 clearly show a high consistency in the results obtained numerically by the original high-dimensional system (1) and by the 1-D reduced system (2). Particularly in Fig. 5D, we further display the steady state *R*_eff_ for the original system (1) and the resilience function for the reduced system (2), respectively. Clearly, the variations of the original system can be captured by the resilience function based on the reduced model. All these confirm that the dimension reduction method is an effective way to quantify the resilience of the coal market networks.

**Fig. 6.**
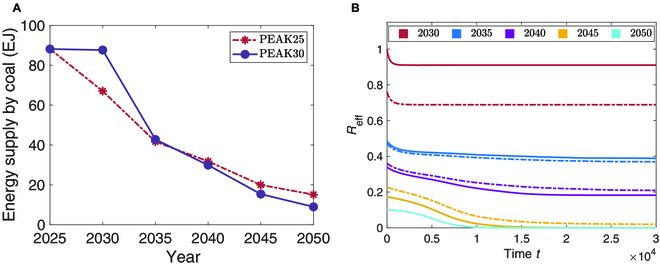
Influence of the energy supply decarbonization. (A) Carbon neutrality-oriented coal supply reduction trajectories under scenarios PEAK25 and PEAK30. (B) The evolution of *R*_eff_ in different scenarios and years. Dashed and solid lines represent scenarios PEAK25 and PEAK30, respectively.

China has consistently emphasized its firm commitment on multiple occasions: striving to reach the peak of carbon dioxide emissions by 2030 and achieve carbon neutrality by 2060. As reported in [3] where carbon neutrality-oriented emission reduction trajectories are given in 3 scenarios, eliminating fossil fuel consumption is one basis of the energy system transformation. The coal supply after reaching its peak in 2025 are shown in Fig. 6A (also reported in Fig. 3 of [3]), which should decrease year by year for achieving carbon neutrality. Here, 2 scenarios are considered that reaching peak in 2025 (PEAK25) and 2030 (PEAK30). The evolutions of the coal production under the designed emission reduction trajectories are displayed in Fig. 6B.

In the simulations, consider the carrying capacity *K* of each coal mine as its maximum allowable coal production capacity, which is regulated by policies. Normalizing the carrying capacity such that *K* = 1 in 2025, and that of each year after 2025 are normalized according to the proportion of their coal supply to that of 2025.

As observed in Fig. 6B, *R*_eff_ converges to stable states in different scenarios. One can see that the smaller values of *K*, the lower the steady output of coal in the market, which means that restricting the maximum allowable production capacity of coal mines can significantly reduce coal supply, thereby reducing carbon emissions. However, it is not conducive to the resilience of the coal network. After 2045, the coal supply is almost less than 20% of that in 2025 as displayed in Fig. 6A, and the steady coal supply tends to zero observed in Fig. 6B, which implies that the resilience of the coal market network is totally lost and it aligns well with the theoretical results in Fig. 2. When the coal supply is at a low level, a further reduction of the maximum allowable production capacity of coal mines may lead to an unexpected collapse of coal supply. Reducing the maximum coal production capacity will inevitable damage the resilience of the coal market network. Thus, for maintaining the energy supply, it is necessary for the Chinese government to make appropriate adjustments of the maximum allowable capacity of coal mines during the process of carbon neutrality.

**Fig. 7.**
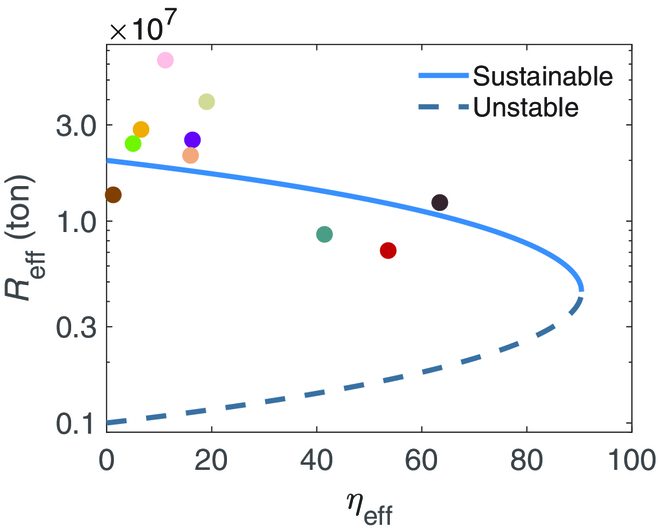
Resilience of China’s coal market network from 2011 to 2020. The bifurcation parameter *η*_eff_ versus the effective annual average output *R*_eff_ of China’s coal market network.

All the above results suggest that network complexity, such as network connectivity and average interaction strength, has significant influence on the resilience of China’s coal market network. Since removal nodes or links can decrease the network connectivity and decreasing the link weights can reduce the average interaction strength among the nodes, reducing the network complexity plays positive roles in promoting the resilience. Interestingly, this viewpoint is contrary to the results reported from the social and economic systems, where amplifying complexity (i.e., especially increasing connectivity) often results in a stronger resilience [26,28]. In fact, increasing the connectivity of the coal market network can cause excessive competition among the coal mines, which naturally yields an undesirable low steady state of coal output. To further demonstrate our viewpoint, using China’s coal transaction data from 2011 to 2020, we calculate and depict in Fig. 7 the effective mean output and the bifurcation parameter *η*_eff_, respectively, for China’s coal market network. The empirical pieces of evidence show that the coal market network with lower connectivity owns relatively higher average output and that almost all empirical data are captured by the resilience function of the 1-D reduced system.

## Conclusion

Coal, as the primary energy source in China, accounts for 75% of the country’s carbon emissions [9]. Faced with the challenge of achieving carbon peak emissions before 2030 and carbon neutrality before 2060, China’s energy consumption is transitioning from fossil-based to nonfossil energy sources. China’s carbon neutrality goals are expected to significantly enhance air quality and safeguard public health [52]. However, the substitution of nonfossil energy for coal is not feasible in the very short term. Consequently, coal will continue to play a pivotal role in China’s economic development and civilization for the next several decades. The policies aimed at decarbonizing energy supply and demand are bound to have a significant impact on the resilience of the coal market. Although numerous studies have explored the risks and resilience of energy supply [43,53], previous research has predominantly treated the energy market as a whole, overlooking its intricate microscopic details. In reality, the coal market is a complex system composed of numerous coal mines. Drawing upon detailed transaction data between coal mines and thermal power plants, we have reconstructed China’s coal market network, thereby enabling an analysis of the resilience of the coal market from a perspective of network science.

Policy interventions and fluctuations in coal demand may result in a loss of resilience of the coal market. Such a loss could lead to coal enterprise bankruptcies and market depression, which in turn could threaten electricity supply and economic development. Consequently, an exploration of the factors that determine the resilience of the coal market is of urgent importance. Inspired by the resilience theory developed for ecosystems, we have proposed a new framework for quantifying the resilience of China’s coal market. The coal market network is characterized by a high-dimensional parameter space, encompassing the intrinsic dynamics of individual coal mines and the complex interactions between them. Consequently, studying its resilience directly is impractical, and causal testing may offer some insights [54]. Our framework, which collapses the high-dimensional coal network onto a 1-D reduced system, enables us to capture some fundamental mechanisms of resilience loss using generic and real coal networks reconstructed from empirical data.

While the model can effectively utilize network topology derived from real data, the parameter settings remain empirical. The challenge lies in obtaining model parameters that better align with real-world conditions based on empirical data. This issue is crucial for ensuring the model’s accuracy and relevance to practical scenarios. The energy structures and markets differ greatly across different countries [55,56]. Therefore, a direction for future research is to improve the model by incorporating a wider range of influencing factors, thereby better capturing the actual situations when applying the model to a greater number of countries.

We have specifically investigated the impact of connectivity, modularity, and heterogeneity within the coal market network, as well as the effects of several realistic perturbations due to changes in energy policies aimed at achieving China’s carbon neutrality target, on the resilience of the coal market network. In previous studies, network connectivity is often reported as a crucial and positive factor in sustaining the resilience of ecosystems [19] and social and economic systems [26,28]. Our research has revealed that the coal market network exhibits low connectivity and a correspondingly low risk of resilience loss. This finding contrasts with the conventional belief that increasing connectivity enhances network resilience [26,28]. Additionally, the coal market network often exhibits low connectivity due to the high transportation costs associated with coal as a bulk commodity, which restricts long-distance coal flows. Moreover, coal mines, particularly small-scale operations, often prefer to sell coal to local thermal power plants. To secure coal supply, thermal power plants often source from multiple coal enterprises, thereby increasing the connectivity of the coal market network. As discussed earlier, this increase in connectivity implies a heightened risk of resilience loss and a reduction in the average coal output of the mines. Therefore, there exists a trade-off between the objectives of thermal power plants, which include enhancing the security of coal supply and improving the resilience of the coal market network.

To mitigate safety hazards in coal production and to advance the low-carbon development strategy, China has closed numerous coal mines with suboptimal production conditions. From a network science perspective, we examined the impact of coal mine closures on the resilience of the coal market network by treating mine closures as the removal of nodes from the network. Our analysis revealed that the closure of small-scale coal mines can enhance the resilience of the coal market. This is attributed to the fact that such closures can sustain the network at a relatively high average output, as demonstrated in Fig. 5A and Fig. S1A.

Furthermore, our analysis has demonstrated that an increase in the intensity of interaction among coal mines can diminish the resilience of the coal market network (as illustrated in Fig. 5C and Fig. S8B). This finding contrasts with the established understanding in other domains, such as labor markets, where greater connectivity is generally associated with increased resilience.

Our findings hold significant implications for the design of energy policy. To achieve the goal of carbon neutrality, decarbonizing the energy supply is indispensable. As indicated by a recent study, the share of renewable energy in primary energy supply is projected to surpass that of fossil fuels by 2050 [3]. However, the transition to a nonfossil energy future is a gradual process, and coal will continue to dominate China’s energy supply for the foreseeable future. Consequently, maintaining the stability of the coal supply is crucial for China’s economic development. Policies that aim to reduce the reliance on coal by restricting the production of all coal mines could inadvertently lead to an undesired low steady state of average output for all mines, which is detrimental to the coal supply. In light of our results, the closure of small-scale coal mines has been shown to significantly enhance the resilience of the coal market. Therefore, a policy that eliminates the production capacity of small-scale mines with outdated technology while allowing the larger enterprises to operate at full capacity could represent an effective approach to reducing the share of coal in the energy mix.

Our investigations have indicated that reducing the connectivity of the coal market network may enhance its resilience. However, the market behavior of thermal power plants, which purchase coal from multiple enterprises to stabilize their thermal coal supply, can lead to an increase in network connectivity, thus intensifying competition within the coal market. Our framework allows for the simulation of a decrease in connectivity by removing links or reducing link weights within the network. Given the uneven geographical distribution of coal resources in China and the recognition that modularity is beneficial for enhancing the resilience of the coal market network, one potential policy intervention could involve restricting long-distance coal trade. Policymakers should also promote low-carbon development strategies, such as the widespread adoption of renewable energy and the continued phase-out of energy-intensive industries, particularly in economically developed regions like the Yangtze River Delta and Pearl River Delta. This approach can enhance the modularity of China’s coal market network. Moreover, intense competition, which corresponds to strong interactions, is detrimental to the resilience of the coal market network. Policy makers should encourage power plants to establish long-term contracts with multiple coal enterprises. This strategy not only reduces connectivity and competition within the coal market network but also mitigates coal prices and production costs for downstream enterprises. Furthermore, the government should incentivize the construction of more thermal power plants in major coal-producing regions and enhance the technology for long-distance electricity transmission. By doing so, cross-regional coal trade can be significantly reduced, thereby diminishing the connectivity of the coal market network.

Our results further indicate that moderately regulating the maximum production capacity of coal mines is an effective means to align with the decarbonization requirements of the energy supply, thus contributing to the achievement of carbon peaking and carbon neutrality. However, this measure inherently compromises the resilience of the coal network, as evidenced in Fig. 6B. Given the significance of the coal mining industry, it is not advisable to abruptly reduce the production capacity of all coal mines. Instead, a comprehensive suite of policies should be implemented concurrently to preserve the resilience of the coal network and ensure China’s energy security.

Overall, China has set forth its carbon neutrality goal and crafted national mitigation strategies. The ongoing phase-out of coal and the expansion of renewable energy are critical to this goal. However, these efforts also pose a near-term threat to China’s energy security, as renewable energy sources are still a long way from fully replacing coal. Therefore, it is crucial to pay greater attention to the impact of energy supply decarbonization on the coal supply. Facing the challenge of carbon neutrality, China’s energy policy must not only adhere to low-carbon emissions requirements but also ensure the security of energy supply. Policymakers must strike a balance between carbon emission reduction and energy security.

## Methods

### Reducing higher-dimensional system into 1-D effective system

Let *f_i_*(*R_j_*) = *θ_ij_R_j_* with *θ_ij_*, the inflow rate parameter. Then, system (1) becomes

$$\begin{array}{ll} \frac{{\mathrm{dR}}_{i}}{\mathrm{dt}}= & -\alpha_{i}A_{i}R_{i}+\left(\alpha_{i}+\frac{\alpha_{i}A_{i}}{K_{i}}\right)R_{i}^{2}-\frac{\alpha_{i}}{K_{i}}R_{i}^{3}\\ & -\delta \sum_{j=1}^{N}\;C_{\mathrm{ij}}\theta_{\mathrm{ij}}R_{i}R_{j}. \end{array}$$ (4)

Note that system (4) is of higher-dimension, containing a large number of interacting components and higher-dimensional parameter space that depends on the node dynamics and the network topologies. Therefore, the classic framework for studying the resilience of lower-dimensional systems cannot be used to analyze the resilience of the higher-dimensional system (4). In light of the framework proposed in Ref. [37], we define an operator by

$$L\left(x\right)=\frac{\frac{1}{N}\sum_{j=1}^{N}\;s_{j}^{\text{out}}x_{j}}{\frac{1}{N}\sum_{j=1}^{N}\;s_{j}^{\text{out}}},$$

where *x* = {*x*_1_, …, *x_N_*} ∈ *R^N^*, $s_{j}^{\text{out}}$ is the out-degree of node *j*. Then, system (4) can be reduced to a 1-D effective system as

$$\begin{array}{ll} \frac{{\mathrm{dR}}_{\text{eff}}}{\mathrm{dt}}= & -L\left(\vec{\alpha }\circ \vec{A}\right)R_{\text{eff}}+L\left(\vec{\alpha }+\frac{\vec{\alpha }\circ \vec{A}}{\vec{K}}\right)R_{\text{eff}}^{2}\\ & -L\left(\frac{\vec{\alpha }}{\vec{K}}\right)R_{\text{eff}}^{3}-{\delta \theta }_{\text{eff}}\beta_{\text{eff}}R_{\text{eff}}^{2}, \end{array}$$

where *R*_eff_ = *L*(*R*) represents the effective mean coal output of the coal market network, *β*_eff_ = *L*(*s*^in^) is an effective control parameter combining the influence of network topologies, and *s*^in^ is the in-degree vector of nodes. Here, we set *θ_ij_* = *θ_j_* and $\theta_{\text{eff}}=L\left(\vec{\theta }\right)$ with $\vec{\theta }=\left\{\theta_{1},\ldots ,\theta_{N}\right\}$. In addition, $\vec{\alpha }\;=\;\left\{\alpha_{1},\ldots ,\alpha_{N}\right\}$, $\vec{A}\;=\;\left\{A_{1},\ldots ,A_{N}\right\}$, and $\vec{K}\;=\;\left\{K_{1},\ldots ,K_{N}\right\}$are the parameter vectors specifying the set parameters characteristics for each node.

Note that $L\left(\vec{a}\circ \vec{b}\right)=L\left(\vec{a}\right)L\left(\vec{b}\right)$, where ∘ is Hadamard’s product. Then, the above equation can be rewritten as

$$\begin{array}{cc} \frac{{\mathrm{dR}}_{\text{eff}}}{\mathrm{dt}}= & -\alpha_{\text{eff}}A_{\text{eff}}R_{\text{eff}}+\left(\alpha_{\text{eff}}+\frac{\alpha_{\text{eff}}A_{\text{eff}}}{K_{\text{eff}}}\right)R_{\text{eff}}^{2}\\ & -\frac{\alpha_{\text{eff}}}{K_{\text{eff}}}R_{\text{eff}}^{3}-{\delta \theta }_{\text{eff}}\beta_{\text{eff}}R_{\text{eff}}^{2}, \end{array}$$

where $\alpha_{\text{eff}}=L\left(\vec{\alpha }\right)$, $A_{\text{eff}}=L\left(\vec{A}\right)$, and $K_{\text{eff}}=L\left(\vec{K}\right)$.

Denote by *η*_eff_ = *θ*_eff _*β*_eff_. Then, we obtain the final form as

$$\frac{{\mathrm{dR}}_{\text{eff}}}{\mathrm{dt}}≜F\left(\eta_{\text{eff}},R_{\text{eff}}\right),$$ (5)

where

Fηeff,Reff=Reff−αeffAeff+αeff+αeffAeffKeff−δηeffReff−αeffKeffReff2. (6)

### Bifurcation analysis of 1-D effective system

Based on the above preliminary introduction and preparations, we firstly analyze the case of fixed points of Eq. 5, which are found by equating *F*(*η*_eff_, *R*_eff_) to zero, namely,

Reff−αeffAeff−αeffKeffReff2+αeff+αeffAeffKeff−δηeffReff=0.

Obviously, the above equation has a trivial solution $R_{\text{eff}}^{*}=0$, representing the depleted state. Besides, to obtain the active state $R_{\text{eff}}^{*}>0$, we let

$$\begin{array}{c} f\left(\eta_{\text{eff}},R_{\text{eff}}\right)=R_{\text{eff}}^{2}-\left[K_{\text{eff}}+A_{\text{eff}}-\frac{\delta K_{\text{eff}}\eta_{\text{eff}}}{\alpha }\right]R_{\text{eff}}+K_{\text{eff}}A_{\text{eff}}. \end{array}$$

Thus, the other equilibria exist for

$$\Delta ={\left(K_{\text{eff}}+A_{\text{eff}}-\frac{\delta K_{\text{eff}}\eta_{\text{eff}}}{\alpha_{\text{eff}}}\right)}^{2}-4K_{\text{eff}}A_{\text{eff}}>0.$$

Thus, the bifurcation point corresponds to the critical value

$$\eta_{\text{eff}}^{c}≜\frac{\alpha_{\text{eff}}}{K_{\text{eff}}\delta_{\text{eff}}}\left(K_{\text{eff}}+A_{\text{eff}}-2\sqrt{K_{\text{eff}}A_{\text{eff}}}\right).$$

As indicated by the above theoretical analysis, Eq. 2 depicts 2 regimes, separated by a critical value $\eta_{\text{eff}}^{c}$. In the first regime of $\eta_{\text{eff}}>\eta_{\text{eff}}^{c}$, the system features a single steady state: $R_{1}≜R_{\text{eff}}^{*}=0$, while, in the second regime of $0<\eta_{\text{eff}}<\eta_{\text{eff}}^{c}$, the system has 3 potential steady states: $R_{1}=R_{\text{eff}}^{*}$, $R_{2}=\frac{1}{2}\left(K_{\text{eff}}+c_{\text{eff}}-\delta \frac{K_{\text{eff}}\eta_{\text{eff}}}{\alpha_{\text{eff}}}\right)-$ $\begin{array}{c} \frac{\sqrt{\Delta }}{2}≜R_{\text{eff}}^{L} \end{array}$, and $R_{3}=\frac{1}{2}\left(K_{\text{eff}}+c_{\text{eff}}-\delta \frac{K_{\text{eff}}\eta_{\text{eff}}}{\alpha_{\text{eff}}}\right)+\frac{\sqrt{\Delta }}{2}≜R_{\text{eff}}^{H}$. In the first regime, the derivative ${\left.\frac{\partial F}{\partial R_{\text{eff}}}\right|}_{R_{\text{eff}}^{*}}<0$ represents a stable nonresilient phase; however, in the second regime, ${\left.\frac{\partial F}{\partial R_{\text{eff}}}\right|}_{R_{\text{eff}}^{*}}<0$ and ${\left.\frac{\partial F}{\partial R_{\text{eff}}}\right|}_{R_{\text{eff}}^{H}}<0$ indicate that the steady states $R_{\text{eff}}^{*,H}$ are stable. The derivative at the intermediate steady state $R_{\text{eff}}^{L}$ satisfies ${\left.\frac{\partial F}{\partial R_{\text{eff}}}\right|}_{R_{\text{eff}}^{L}}>0$, so that $R_{\text{eff}}^{L}$ is unstable. Hence, a fluctuation easily drives the system below $R_{\text{eff}}^{L}$ to the state of resilience loss.

### Generation of modular networks

We generate homogeneous modular networks as follows. For a modular network with *N* nodes and *m* modules, all modules are constructed using the ER networks with the same network size and local connectivity *c_l_*. Then, connecting any 2 nodes from different modules takes a probability of *c_g_*.

### Reconstruction of China’s coal market network

In China, 72.5% coal is used for electricity generation in 2020. Thus, the coal market network can be naturally reconstructed by the transaction data between the coal mines and the power plants. Two coal mines are said to be connected if they supply the thermal coal to the same power plant. In this way, the coal market network is the projection network of the bipartite coal-electricity networks (see Fig. 1). Although there are more than 1,000 coal mines in China, most of them are small-sized and the top 30 coal enterprises account for more than 60% of the total coal output.

Moreover, it is difficult to obtain the transaction data of the small-sized coal mines.

Thus, in Fig. 1, we reconstructed China’s coal market network, which consists of 100 coal mines located mainly in the 3 provinces of Shanxi, Inner Mongolia, and Shannxi. Actually, the enterprises in these 3 provinces account for over 60% total coal output of China. When we consider the influence of some realistic perturbations on the resilience (see Fig. 5), the coal networks in 2020 was used to test the validity of the 1-D reduced system.

The coal market networks, which are reconstructed from the history transaction data from 2011 to 2020, allow us to further demonstrate the efficacy of the theoretical framework we established in Results (refer to Fig. 7).

## Data Availability

The datasets generated during and/or analyzed during the current study are all available from the corresponding authors upon request. All relevant computer codes are available from the authors upon request. Supplementary information is available for this paper.

## Supplementary Materials

Supplementary 1 Sections S1 to S5 Figs. S1 to S8
