# Supplementary material for: Quantifying the Resilience of Coal Energy Supply in China Toward Carbon Neutrality
Source: Research (Wash D C). 2024 Jul 16;7:0398. doi: 10.34133/research.0398 (PMC11249919; doi:10.34133/research.0398)
Supplement: Supplementary 1 — Sections S1 to S5 Figs. S1 to S8 [file research.0398.f1.pdf]

Supplementary Information for

Quantifying the resilience of coal energy supply in china towards carbon neutrality*

*E-mails: yzsung@gmail.com, wlin@fudan.edu.cn, f.zhou@cumt.edu.cn

I. NOTATIONS

- Acronyms: "CO_2" for carbon dioxide, "1D" for one-dimensional, and COP28 for the 28th Conference of the Parties to the United Nations Framework Convention on Climate Change.
- N represents the number of coal mines or coal enterprises.
- Indices i and j refer to specific coal mines, with $R_i(t)$ denoting the time-dependent coal production.
- The connections among coal mines are represented as a weighted adjacency matrix C with items C_{ij}, where $C_{ij} > 0$ if coal mines i and j supply coal for a same thermal power plant, otherwise; $C_{ij} = 0$.
- Parameters $\alpha_i > 0$, A_i and K_i characterize the intrinsic growth rate, Allee effect, and carrying capacity of each coal mine, respectively. The parameterδ quantifies the strength of network interaction. The function f_i illustrates how coal mine j influences i through market competition, and θ_{ij} represents the inflow rate parameter.
- R_{eff} is the effective average coal production of the whole coal market, $\eta_{\text{eff}} = \theta_{\text{eff}}\beta_{\text{eff}}$ with the macroscopic parameter β_{eff} characterizing the combined effect of the microscopic description C_{ij} of the inter competition.
- α_{eff}, A_{eff}, K_{eff} and θ_{eff} account for the effective growth rate, the Allee effect, the carrying capacity, and the inflow rate, respectively, when averaged across the network structure.

II. DATA FOR QUANTIFYING COAL MARKET NETWORKS

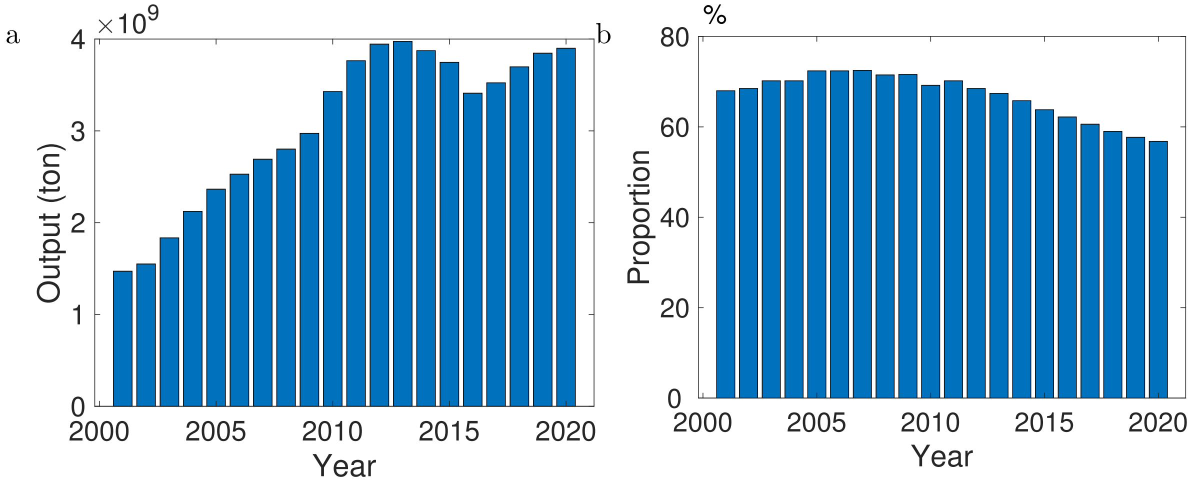

FIG. S1: **Total output of coal and share of coal in China's energy consumption from 2000 to 2020.** (a) Share of coal in China's energy consumption. (b) Total output of coal in China. Here, the total coal output reach its peak in 2013, while the share of coal in China's energy consumption gradually decreased from 72.8% in 2007 to 56.8% in 2020.

III. INFLUENCE OF CONNECTIVITY ON THE RESILIENCE OF COAL MARKET NETWORK WITH WS AND BA TOPOLOGIES

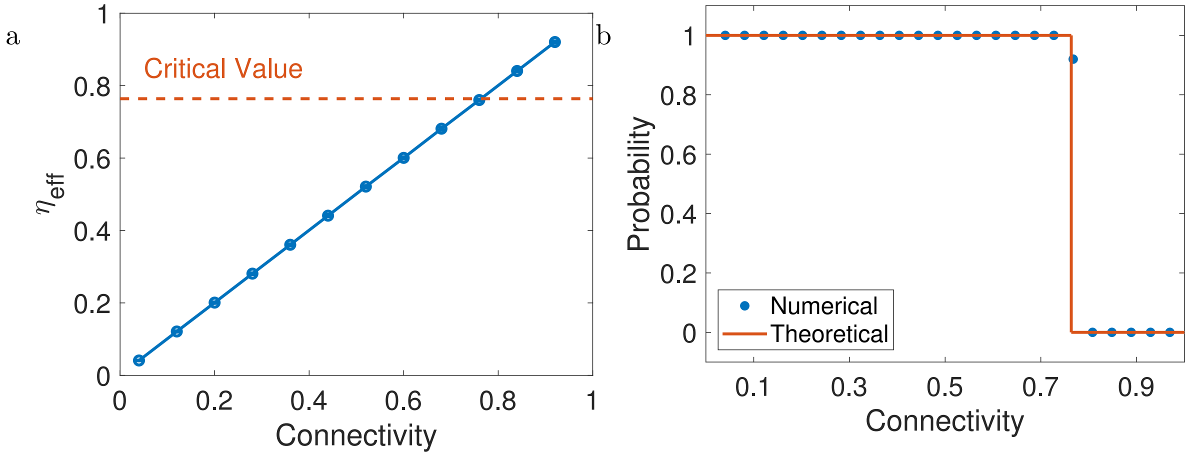

FIG. S2: **Influence of connectivity on resilience of coal market network with WS small-world topologies.** We take $\alpha = 0.5$, $A = 0.2$, $K = 1.0$, $\delta = 0.2$, and $\theta = 0.05$, which gives the critical value $\eta^c_{\mathrm{eff}} = 0.76$ for the resilience loss of the reduced system (2) in the main text. Using the method articulated in [1], we generated WS small-world networks with different rewiring probability β and a uniform network size $N = 100$. (a) The bifurcation parameter η_{eff} increases monotonically with the rewiring probability β. (b) The probability that system (1) exhibits a sustainable state as the function of the rewiring probability β. The other parameters are the same as those used in Fig. 2 of the main text. The results are obtained through averaging 100 realizations and the initial states are randomly-selected near the steady state R^H_{eff}.

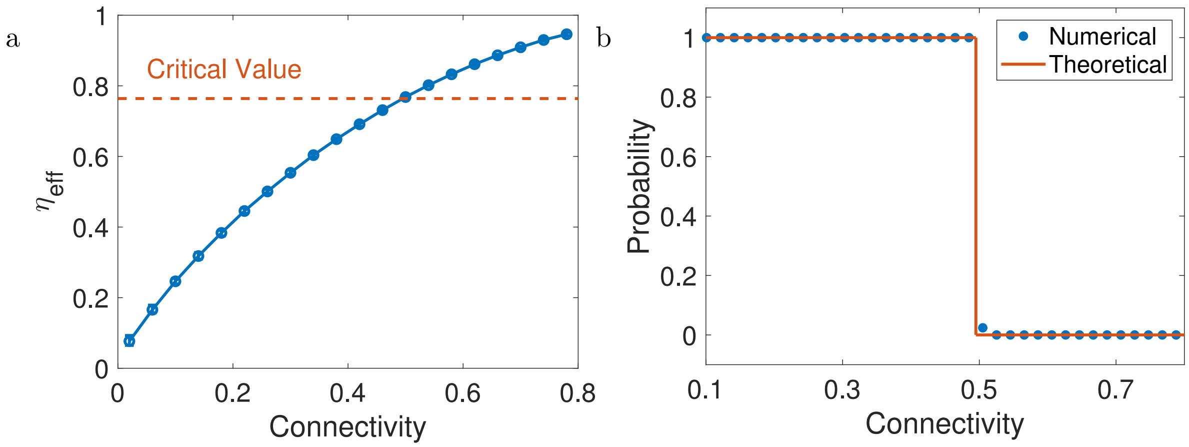

FIG. S3: **Influence of connectivity on resilience of coal market network with BA scale-free topologies.** Here, we take $\alpha = 0.5$, $A = 0.2$, $K = 1.0$, $\delta = 0.2$, and $\theta = 0.05$, which yields the critical value $\eta_{\text{eff}}^{c} = 0.76$ for the resilience loss of the reduced system (2) in the main text. Using the method articulated in [2], we generated BA scale-free networks with different degree exponents γ_d and a uniform network size $N = 100$. (a) The bifurcation parameter η_{eff} increases monotonically with the degree exponent γ_d. (b) The probability that system (1) exhibits a sustainable state as the function of the degree exponents γ_d. The other parameters are the same as those used in Fig. 2 of the main text. The results are obtained through averaging 100 realizations and the initial states are randomly-selected near the steady state R_{eff}^{H}.

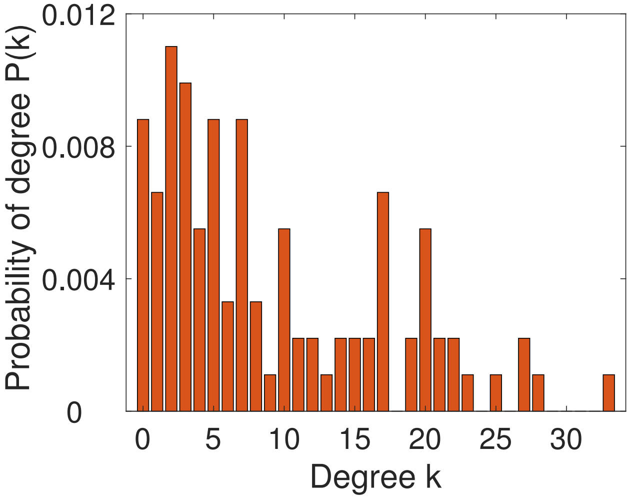

FIG. S4: An empirical distribution of the network degrees for China's coal market network shown in Fig. 1b of the main text.

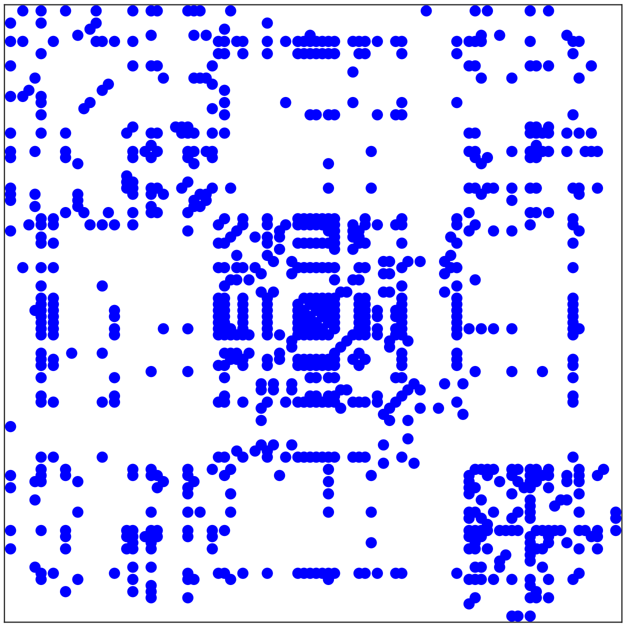

FIG. S5: An empirical adjacency matrix of China's coal market network shown in Fig. 1b of the main text.

IV. DEGREE DISTRIBUTION AND MODULARITY OF CHINA'S COAL MARKET NETWORKS

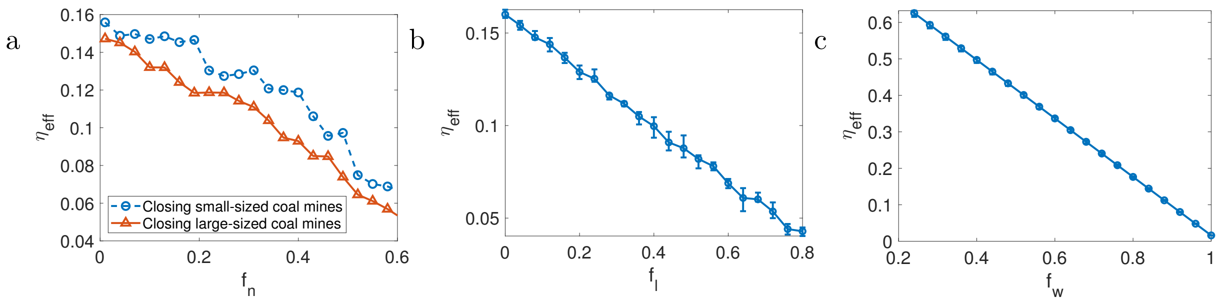

FIG. S6: **Influence of external disturbances on bifurcation parameter $\eta_{\rm eff}$ of China's coal market network.** Here, the values of $\eta_{\rm eff}$ as functions of different fractions: (a) f_n, the closing fraction of large-sized coal mines (squares) and small-sized coal mines (circles), (b) f_l, the removal fraction of the links in the network, and (c) f_w, the reduction fraction of the weights in the network. The parameters are the same as those used in Fig. 6 of the main text.

V. INFLUENCE OF EXTERNAL DISTURBANCES ON BIFURCATION PARAMETER AND RESILIENCE OF EMPIRICAL AN ER NETWORKS

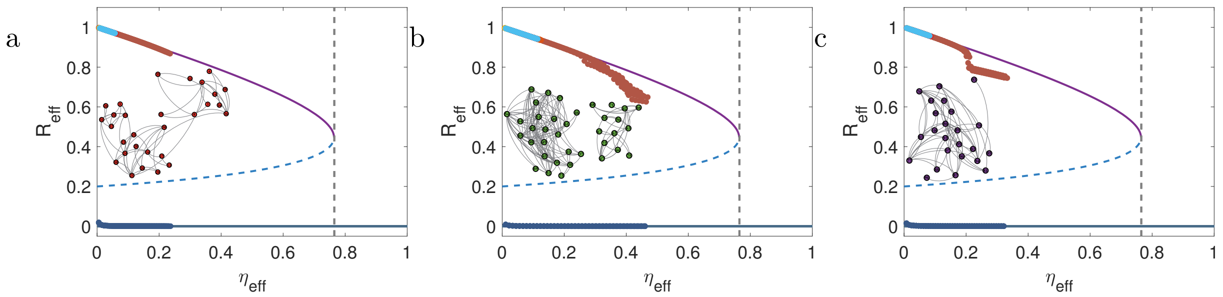

FIG. S7: **Influence of external disturbances on empirical coal market networks of three provinces.** Here, the points of $(\eta_{\rm eff}, R_{\rm eff})$ are plotted, respectively, for Shanxi (a), Inner Mongolia (b) and Shannxi (c) with external disturbances. The parameters are the same as those used in Fig. 3 of the main text.

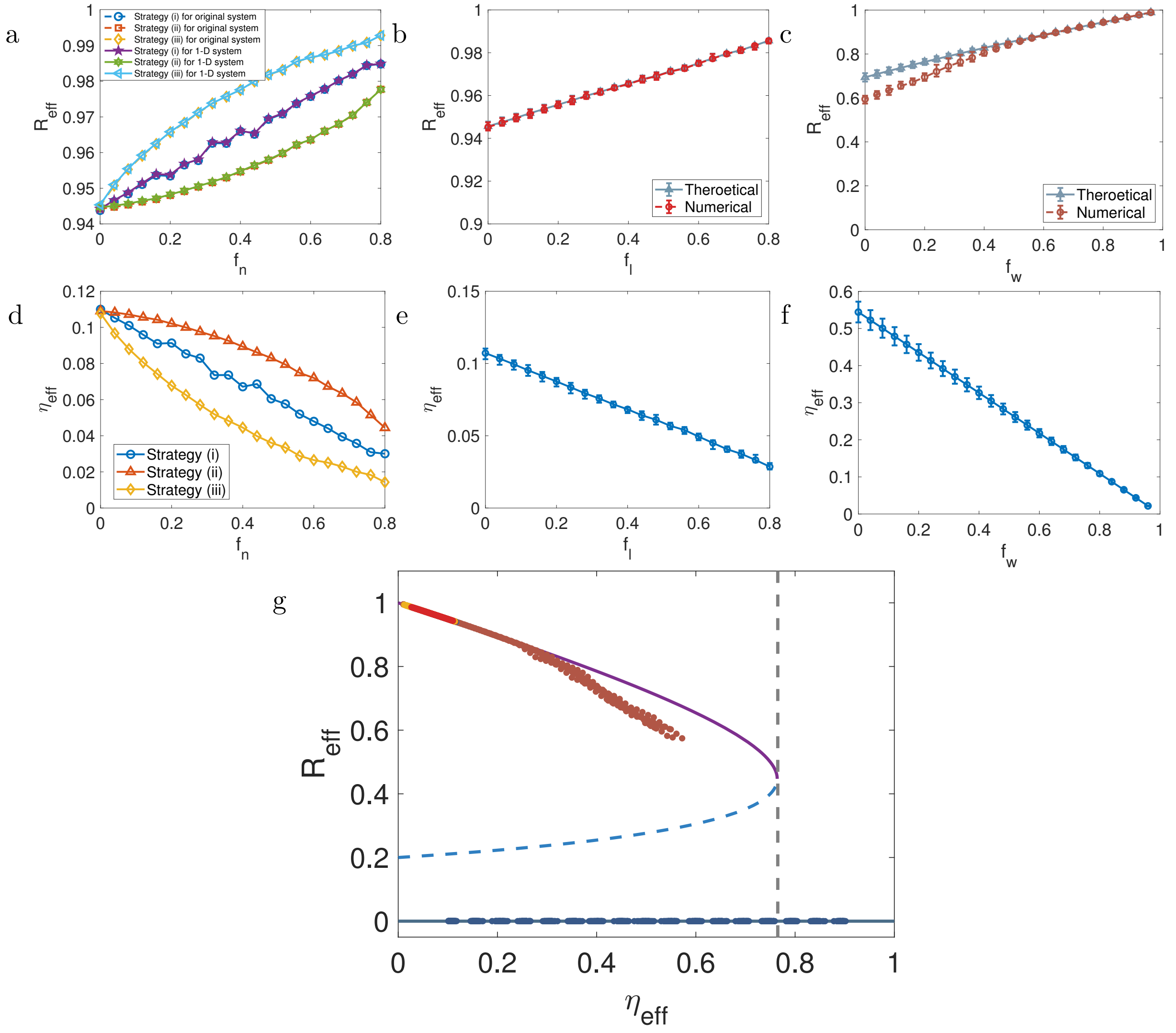

FIG. S8: **Influence of external disturbances on bifurcation parameter R_{eff} and η_{eff} with ER networks.** Here, the values of R_{eff} and η_{eff} as functions of different fractions: (a,d) f_n , the closing fraction of the coal mines, (b,e) f_l, the cancelling fraction of the transactions between the coal mines and the thermal power plants, and (c,f) f_w, the fraction to which all weights on average are decreased, mimicking the market depression. Here, the parameters are the same as those used in Fig. 3 of the main text. (g) the points of $(\eta_{\text{eff}}, R_{\text{eff}})$ corresponding to (a-f) are plotted.

[1] Watts D J, Strogatz S H. Collective dynamics of ‘small-world’networks[J]. Nature, 1998, 393(6684): 440-442.

[2] Catanzaro M, Boguná M, Pastor-Satorras R. Generation of uncorrelated random scale-free networks[J]. Physical Review E, 2005, 71(2): 027103.
